# Phase I dose-escalation study of helical intensity-modulated radiotherapy-based stereotactic body radiotherapy for hepatocellular carcinoma

**DOI:** 10.18632/oncotarget.9450

**Published:** 2016-05-18

**Authors:** Jun Won Kim, Jinsil Seong, Ik Jae Lee, Joong Yeol Woo, Kwang-Hyub Han

**Affiliations:** ^1^ Department of Radiation Oncology, Gangnam Severance Hospital, Yonsei University College of Medicine, Seoul, Korea; ^2^ Department of Radiation Oncology, Yonsei Cancer Hospital, Yonsei University College of Medicine, Seoul, Korea; ^3^ Department of Internal Medicine, Severance Hospital, Yonsei University College of Medicine, Seoul, Korea

**Keywords:** stereotactic body radiotherapy, hepatocellular carcinoma, dose escalation, intensity-modulated radiotherapy

## Abstract

**Background:**

Phase I trial was conducted to determine feasibility and toxicity of helical intensity-modulated radiotherapy (IMRT)-based stereotactic body radiotherapy (SBRT) for hepatocellular carcinoma (HCC).

**Results:**

Eighteen patients (22 lesions) were enrolled. With no DLT at 52 Gy (13 Gy/fraction), protocol was amended for further escalation to 60 Gy (15 Gy/fraction). Radiologic complete response rate was 88.9%. Two outfield intrahepatic, 2 distant, 4 concurrent local and outfield, and 1 concurrent local, outfield and distant failures (no local failure at dose levels 3–4) occurred. The worst toxicity was grade 3 hematologic in five patients, with no gastrointestinal toxicity > grade 1. At median follow-up of 28 months for living patients, 2-year local control, progression-free (PFS), and overall survival rates were 71.3%, 49.4% and 69.3%, respectively. Multi-segmental recurrences prior to SBRT was independent prognostic factor for PFS (*p* = 0.033).

**Materials and Methods:**

Eligible patients had Child-Pugh's class A or B, unresectable HCC, ≤ 3 lesions, and cumulative tumor diameter ≤ 6 cm. Starting at 36 Gy in four fractions, dose was escalated with 2 Gy/fraction per dose-level. CTCAE v 3.0 ≥ grade 3 gastrointestinal toxicity and radiation induced liver disease defined dose-limiting toxicity (DLT).

**Conclusions:**

Helical IMRT-based SBRT was tolerable and showed encouraging results. Confirmatory phase II trial is underway.

## INTRODUCTION

The most common etiology for hepatocellular carcinoma (HCC) in Korea is viral infection (hepatitis B and C), and a large proportion of newly diagnosed HCC accompany advanced cirrhosis [1]. Surgical resection and percutaneous ablation can provide long-term overall survival [2]; however, eligibility for these curative procedures is limited by preexisting conditions including hepatic dysfunction, tumor number and size, and vascular invasion. Stereotactic body radiotherapy (SBRT) has shown high rates of local control for primary and metastatic liver cancers [3–5]. In SBRT, a biologically equivalent dose > 100 Gy is delivered using highly conformal, hypofractionated radiation in 2-5 fractions [6], and this technique requires precision targeting and reproducibility in treatment setting. Given the steep dose fall-off and reduced number of fractions, the risk of local failure and normal tissue injury due to geometric miss is high with SBRT. Thus, major challenges in achieving safe and accurate SBRT for intrahepatic tumors include defining and limiting respiratory liver motion during treatment and providing accurate daily image-guidance.

Helical Tomotherapy (HT) (Accuray, Madison, WI) is well-suited for delivering SBRT. The use of up to 51 beam angles for treatment planning allows for highly conformal dose distributions with improved sparing of normal tissues compared to more conventional 3D plans [7]. HT-based SBRT is often utilized for treating brain [8] or spine lesions [9]; however, its role in treating intrahepatic tumors has not been explored. HT is effective in treating multiple targets simultaneously, and we have previously shown its adequacy for multiple metastatic tumors [10]. The ability of HT to target multiple lesions simultaneously has been demonstrated for intrahepatic tumors [11] as well, and this is an important ability for liver SBRT, because most studies allowed patients with up to three intrahepatic lesions for eligibility [12, 13].

Image guidance for HT is provided via CT detector mounted opposite the radiation source, which is used for megavoltage CT (MVCT) imaging [14]. A common problem in using MVCT for image guidance is the low contrast resolution observed with the MVCT imaging system relative to kilovoltage (kV) CT [15], particularly when localizing treatment sites in the abdomen. However, the contour of the entire liver can be a surrogate, and rigid liver-to-liver registration using MVCT in combination with respiration control can be an effective image-guidance system for liver tumors.

We have been using HT with MVCT image guidance for treating locally advanced HCC at our institution since 2006 [16]. We report the results of a phase I dose escalation trial that was designed to determine the feasibility and toxicity of helical intensity-modulated radiotherapy (IMRT)-based SBRT for primary HCC.

## RESULTS

### Patients

From March 2012 to April 2014, 18 patients with 22 lesions were enrolled. Table 1 shows the demographic and treatment data. All patients had Child-Turcotte-Pugh (CTP) A (score 5 in 17 patients and 6 in one patient), and no patient had portal vein tumor thrombosis. The median cumulative tumor diameter was 2.05 cm (range 1.0–4.4 cm). The dose was initially escalated to 52 Gy (13 Gy/fraction) without DLT. The protocol was amended for a further escalation to 60 Gy (15 Gy/fraction). The total number of patients analyzed in this study included additional patients enrolled in dose levels 1 and 3 while the amended protocols were being approved. Table 2 shows the dosimetric parameters from the radiotherapy planning. The median value for PTV was 79.9 cc (8.16 to 225.3 cc). The median normal liver volume was 1124 cc (801 to 1736 cc), and the median value of the mean dose to normal liver was 9.7 Gy (3.0 to 14.3 Gy).

**Table 1:** Demographic and treatment data (*n* = 18)

Characteristics		No. of patients (%)
Sex	Female : Male	4 : 14 (22.2 : 77.8)
Age		Median 59.5 years (range 42–83)
Hepatitis etiology	B	14 (77.8)
	C	1 (5.5)
	nonB/nonC	3 (16.7)
Previous treatments	None	3 (16.7)
	Multiple TACE	6 (33.3)
	Multiple TACE+RFA	4 (22.2)
	Multi-modality^*^	5 (27.8)
Hepatic segment with recurrence	Single	8 (44.4)
	Multiple	10 (55.6)
Child-Pugh Score	A (5)	17 (94.4)
	A (6)	1 (5.6)
AFP > 9 ng/ml at RT		11 (61.1)
PIVKA > 35 mIU/ml at RT		10 (55.6)
Portal vein thrombosis	No	18 (100)
Number of lesions	1	15 (83.3)
	2	2 (11.1)
	3	1 (5.6)
Maximum tumor diameter		Median 1.95 cm (range 1.0–3.3)
Cumulative tumor diameter		Median 2.05 cm (range 1.0–4.4)
Dose per fraction/total dose	9 Gy/36 Gy	4 (22.2)
	11 Gy/44 Gy	3 (16.7)
	13 Gy/52 Gy	8 (44.4)
	15 Gy/60 Gy^†^	3 (16.7)

* Resection + TACE + RFA (*n* = 2), resection + TACE + sorafenib (*n* = 1), TACE + hepatic arterial chemotherapy + RFA (*n* = 1), and TACE + sorafenib + hepatic arterial chemotherapy (*n* = 1).

† Dose level 4 (15 Gy × 4 fractions) was added after no DLT was observed at level 3.

Abbreviations: RFA = Radiofrequency ablation; TACE = Transarterial chemoembolization; AFP = Alpha-feto protein; PIVKA = Proteins induced by vitamin K absence or antagonist-II; UICC = International union against cancer.

**Table 2:** Dosimetric parameters

PTV volume	Median 79.9 cc (range 8.2–225.3)
Volume of normal liver	Median 1124 cc (range 801–1736)
Mean dose to normal liver	Median 9.7 Gy (range 3.0–14.3)
Dose to 700 cc normal liver	Median 2.65 Gy (range 0.5–10)
Volume of normal liver receiving < 2.5 Gy	Median 800 cc (range 208–1233)
< 5.0 Gy	Median 582 cc (range 160–1074)
< 7.5 Gy	Median 488 cc (range 131–915)
< 10.0 Gy	Median 391 cc (range 101–700)
< 12.5 Gy	Median 311 cc (range 89–533)
< 15.0 Gy	Median 223 cc (range 65–402)
Max dose to bowel/stomach	Median 14.3 Gy (range 0.1–26.4)
Max dose to spinal cord	Median 11.0 Gy (range 4.8–19.5)

Abbreviations: PTV = Planning target volume.

### Toxicity

SBRT was well tolerated, with no DLT observed at any level. Table 3 shows toxicities that worsened from pre-SBRT (baseline) conditions, within the first 3 months after SBRT or prior to salvage treatment in case of treatment failure. The worst liver toxicity was grade 2 hyperbilirubinemia in one patient, and no GI toxicity greater than grade 1 occurred. Grade 3 leukocytopenia and thrombocytopenia developed in two and five patients, respectively. Six of these patients had grade 2 and one had grade 1 hematologic events prior to SBRT. Grade 2 radiation pneumonitis occurred in one patient with HCC in segment 8.

**Table 3:** Treatment related toxicities within 6 months after SBRT

Toxicity		CTCAE v3.0 grade		
		1	2	3
Liver function	AST	7	0	0
	ALT	4	0	0
	Albumin	0	1	0
	ALP	1	0	0
	Bilirubin	0	1	0
	INR	1	0	0
Hematologic	Leukocytes	6	7^*^	2^†^
	Hemoglobin	1	0	0
	Platelets	2	1^‡^	5^§^
Gastrointestinal	Anorexia	3	0	0
	Nausea	1	0	0
Other	Fatigue	4	1	0
	Pain	1	0	0
Pulmonary	RT pneumonitis	6	1	0

Worsening toxicities due to SBRT are recorded; toxicities due to salvage treatment after treatment failure are not recorded.

* Four patients had grade 1 leukocytopenia prior to SBRT.

† Two patients had grade 2 leukocytopenia prior to SBRT.

‡ One patient had grade 1 thrombocytopenia prior to SBRT.

§ Four patients had grade 2 and one patient had grade 1 thrombocytopenia prior to SBRT.

Abbreviations: CTCAE = Common terminology criteria for adverse events; AST = Aspartate Aminotransferase; ALT = Alanine aminotransferase; ALP = Alkaline phosphatase; INR = International normalization ratio.

### Response rate and tumor control

**Figure 1:**
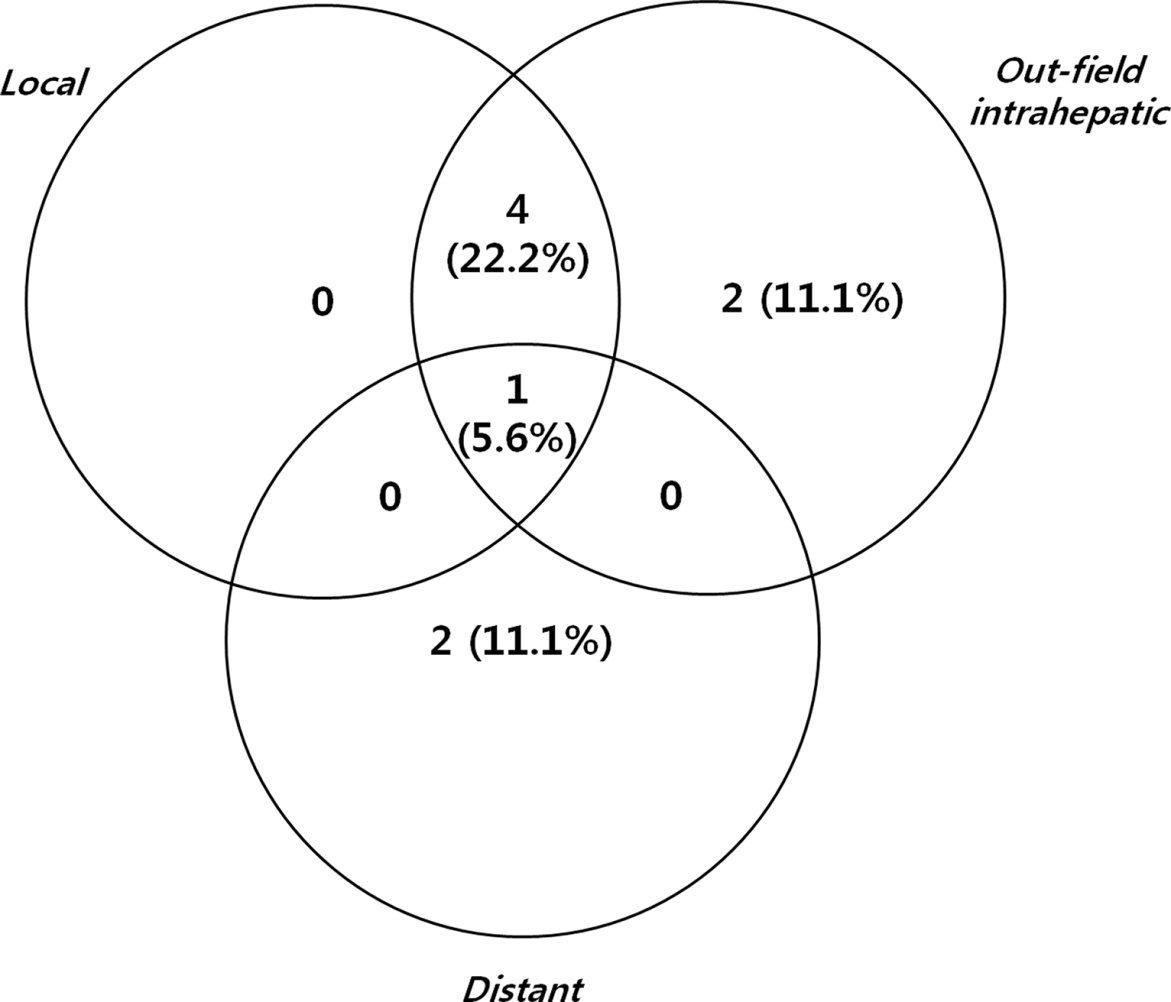
Patterns of all failure

A radiologic CR was achieved in 16 patients (88.9%) with a median time to a radiologic CR of 6.0 months (range 0.7-12.3 months). One patient at dose level 1 achieved a pathologic CR, which was confirmed at liver transplantation 14 months after SBRT. Nine patients experienced disease progression: 2 outfield intrahepatic, 2 distant, 4 local and outfield, and 1 local, outfield and distant failures (Figure 1). At dose level 1, one outfield intrahepatic failure, 1 concurrent local and outfield, and 1 outfield failure followed by local failure were observed. At dose level 2, one local failure followed by outfield failure and 1 lung metastasis were observed. At dose level 3, one outfield failure and 1 lung metastasis were observed, and another patient experienced outfield failure followed by local failure which was due to tumor progression into the treated area. At dose level 4, one patient experienced concurrent outfield failure and lung metastasis followed by local failure which was due to rapid tumor progression into the treated area.

### Survival and prognostic factors

**Figure 2:**
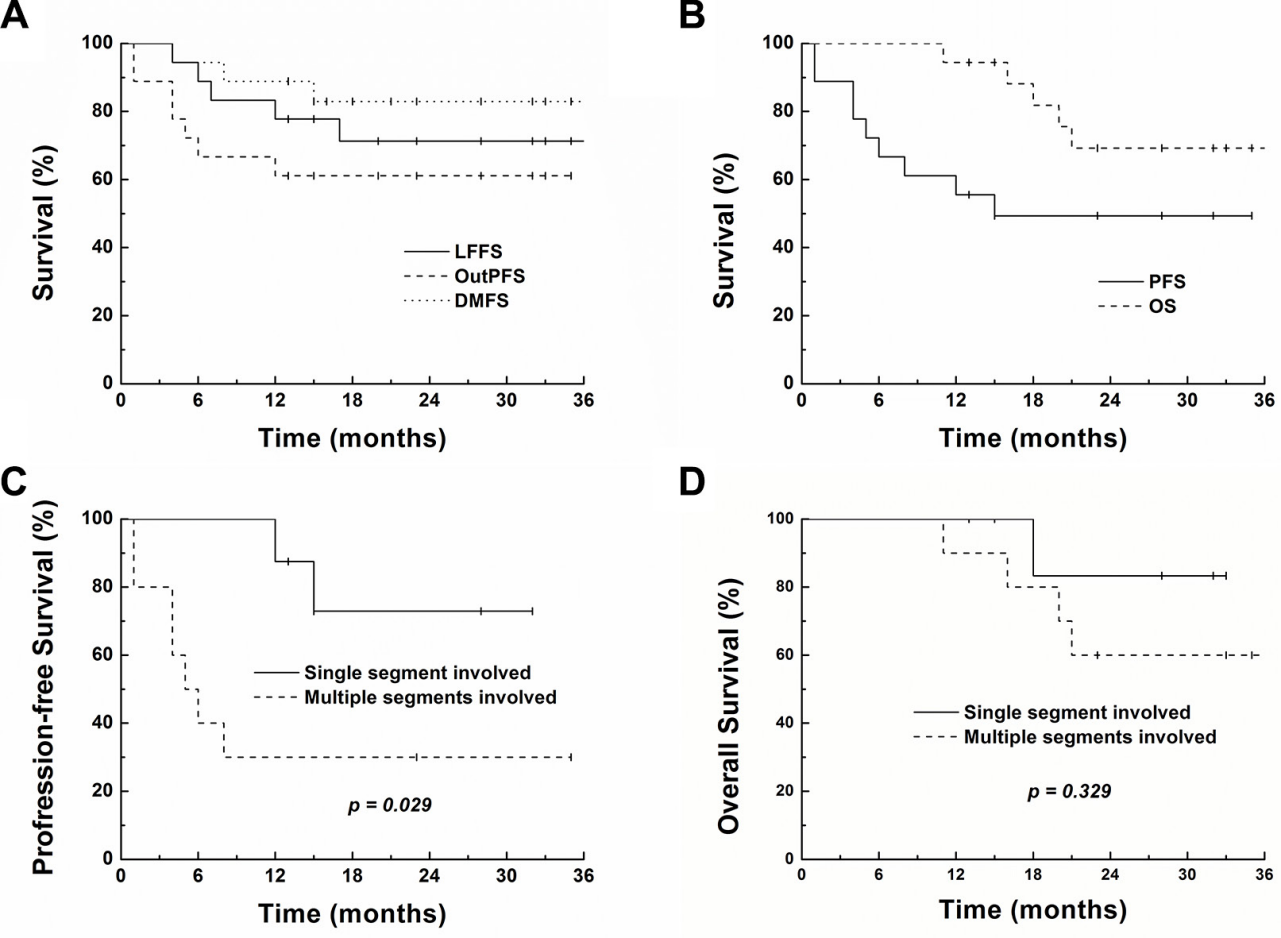
Survival curves showing local failure-free (LFFS), intrahepatic out-field progression-free (OutPFS), distant metastasis-free (DMFS), progression-free (PFS) and overall survival (OS) for all patients (4A and 4B) PFS and OS of the patients with (*n* = 10) and without multi-segment recurrences (*n* = 8) are shown in Figure 4C and 4D.

At a median follow-up of 23 months (range 11–38 months) for all patients and 28 months (range 13–38 months) for living patients, the 1- and 2-year local control rates were 77.8% and 71.3%, outfield intrahepatic progression-free survival rates were 61.1% and 61.1%, distant metastasis-free survival rates were 88.9% and 83.0%, progression-free survival (PFS) rates were 55.6% and 49.4%, and overall survival rates were 94.4% and 69.3%, respectively (Figure 2A and 2B). Table 4 shows the results of univariate and multivariate analyses of the clinical factors influencing PFS. Multi-segmental recurrences prior to SBRT showed a significant correlation with poor PFS rates in univariate (*p* = 0.029) and multivariate analyses (*p* = 0.033). PFS and OS of the patients with (*n* = 10) and without multi-segment recurrences (*n* = 8) are shown in Figure 2C and 2D. Seven of the 10 patients with multi-segmental recurrences showed failures after SBRT: 1 local, 4 out-field intrahepatic, 1 lung metastasis, and 1 synchronous outfield intrahepatic and lung metastasis.

**Table 4:** Factors influencing progression-free survival

Prognostic factors	No of Pts (%)	Univariate analysis			Multivariate analysis		
		2-yr PFS (%)	95% CI	*P*	RR	95% CI	*P*
Dose level							
3–4	11 (61)	63.6	35–92	0.209	2.425	0.47–12.6	0.293
1–2	7 (39)	28.6	0–62				
Age							
< 60	9 (50)	44.4	12–77	0.580			
≥ 60	9 (50)	53.3	19–87				
Etiology							
Other	4 (22)	50.0	1–99	0.694			
HBV	14 (78)	49.0	22–76				
Pre-RT AFP							
< 9 ng/ml	6 (33)	25.0	0–65	0.389			
≥ 9 ng/ml	12 (67)	58.3	30–86				
Cumul diameter							
< 3 cm	13 (72)	61.5	35–87	0.100	4.003	0.42–38.3	0.229
≥ 3 cm	5 (38)	20.0	0–55				
Multiplicity							
No	15 (83)	52.5	27–788	0.289	0.698	0.06–8.7	0.780
Yes	3 (17)	33.3	0–87				
Multi-segments							
No	8 (44)	72.9	41–100	0.029	8.561	1.20–61.3	0.033
Yes	10 (56)	30.0	2–58				

Abbreviations: RR = Relative risk; HBV = Hepatitis B virus.

## DISCUSSION

Challenges in treating intrahepatic tumors with RT include defining and limiting respiratory liver motion, accurate delineation of hypovascular tumors, minimizing intrafractional- and interfractional uncertainties, and poor resolution of tumor in x-ray images used for image guidance. A simple method to overcome these limitations is increasing the PTV margins; however, without effective management of liver motion, the volume of non-target liver will increase, hence increasing hepatic toxicity. Abdominal compression is a simple and effective method of reducing diaphragmatic motion. Using gold fiducial markers, Wunderlink et al. showed that abdominal compression was effective in reducing liver tumor motion, yielding small and reproducible excursions in three dimensions [17]. Using rigid liver-to-liver registration of cone beam CT (CBCT) to planning CT, Eccles et al. showed that interfraction liver deformations and GTV displacement in patients undergoing SBRT with abdominal compression were small in most patients [18].

**Figure 3:**
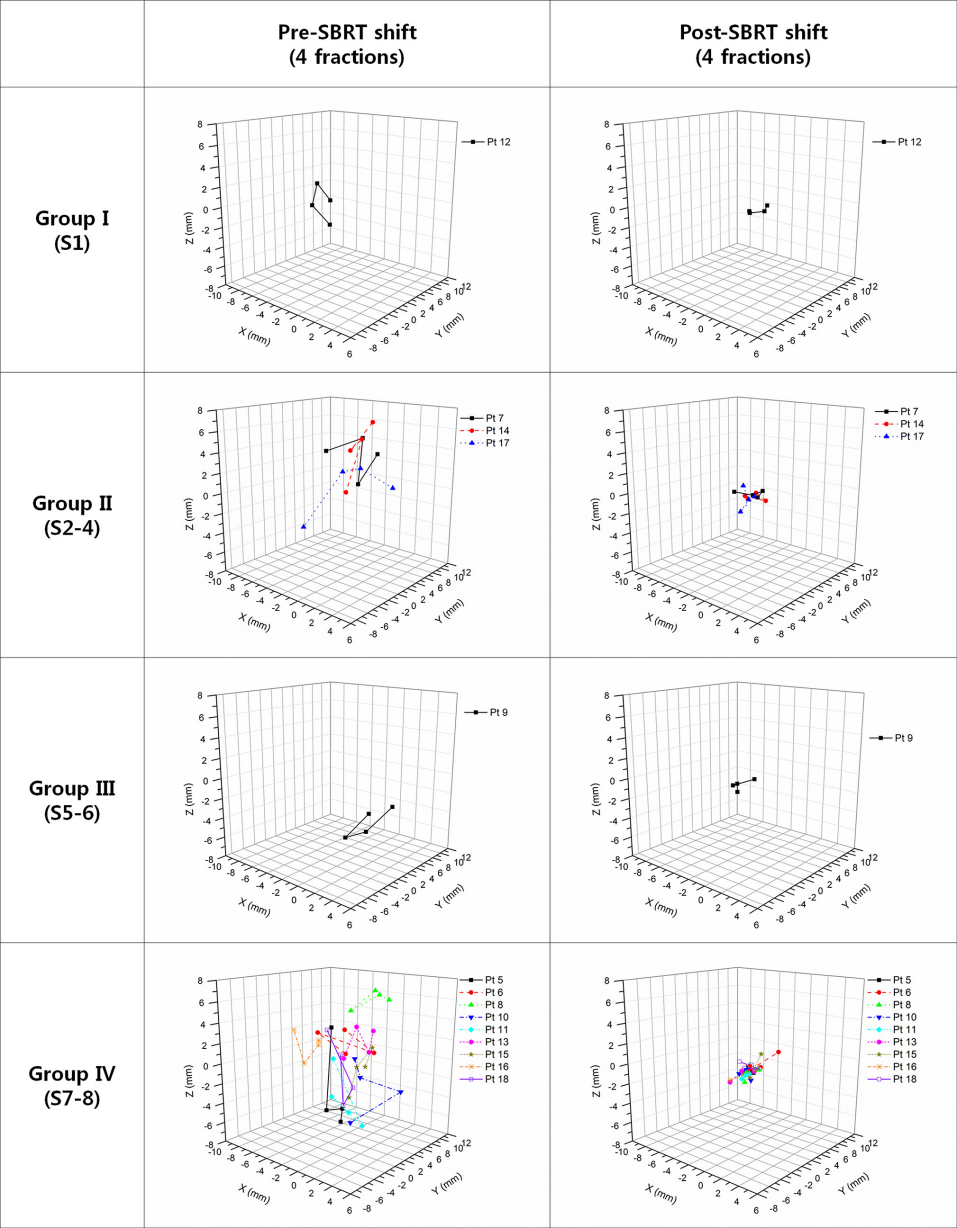
Comparison of pre- and post-treatment setup corrections for 4 fractions Patients are grouped according to tumor location (Group I: segment 1; Group II: segments 2, 3, and 4; Group III: segments 5 and 6; and Group IV: segments 7 and 8).

Fiducial markers have the advantage of being visible on x-ray images and fluoroscopy loops, hence increasing targeting accuracy in image-guided SBRT for intrahepatic tumors. However, fiducial placement carries risks specific to markers including migration, additional costs, and imaging artifacts on CT. Procedure-related risks include pain, pneumothorax, hemothorax, perforation of non-target organs, infection, and tumor seeding. These risks can increase substantially with insertion of multiple markers [19]. An alternative is to use the contour of the entire liver as a surrogate. HT uses onboard MVCT for image guidance. Intrahepatic tumors are known to be poorly visible in MVCT as well as in CBCT images. Since installation of HT at our institution in 2006, we have been using rigid liver-to-liver registration of MVCT to planning CT for treatment with HT [20]. Slow acquisition of MVCT may be a disadvantage; however, abdominal compression reducing breathing motion to < 5 mm and liver-to-liver registration in the 3-dimensional view improves accuracy of image-guidance [21]. Comparison of pre- and post-treatment setup corrections in the current study has shown MVCT is an adequate tool for image-guidance in treating hepatic tumors (Table 5 and Figure 3). Another advantage of HT is its effectiveness in treating multiple targets simultaneously [11], and potential benefits include reduced time for treatment set-up and delivery and increased patient compliance.

**Table 5:** Comparison of pre- and post-treatment setup corrections

Location of tumor	Pre-SBRT displacement R^*^ (average ± SD, mm)	Post-SBRT displacement R^*^ (average ± SD, mm)
Group I (segment 1), *n* = 1	3.9 ± 1.2	1.1 ± 0.7
Group II (segments 2–4), *n* = 3	6.3 ± 2.8	1.5 ± 1.0
Group III (segments 5–6), *n* = 1	7.5 ± 1.3	2.6 ± 0.8
Group IV (segments 7–8), *n* = 10	5.0 ± 2.0	1.6 ± 1.1
All groups, *n* = 14	5.4 ± 2.3	1.6 ± 1.1

* $\text{R}=\sqrt{{\text{X}}^{2}+{\text{Y}}^{2}+{\text{Z}}^{2}}$

Our results suggest that helical IMRT-based SBRT is an effective tool for local control of up to three HCCs targeted simultaneously, provided that an adequate dose of radiation is delivered. Another contributing factor for improving local control may be the added margin for microscopic satellite lesions. Clinicopathologic studies have shown that microscopic satellite lesions of HCC can be detected 5–10 mm around the gross tumor [22, 23]; thus, providing adequate margins is an important but often overlooked issue. Previous dose escalation studies defined CTV as GTV [12] or ITV [13] without an additional margin, whereas we added a 5 mm margin to ITV to define CTV in order to cover microscopic satellite lesions. Extra measures including the use of 4D CT and abdominal compression allowed safe incorporation of the additional margin without increasing PTV substantially.

DLT was not reached even after the dose was escalated to a higher level than initially planned. Our results suggest that a further dose escalation may be possible if eligibility criteria are strictly met. Local control of HCCs, which are relatively radiosensitive, seems achievable at dose level 3; however patients with intrahepatic cholangiocarcinoma and metastatic tumors such as those from colorectal cancer may require a higher dose for prolonged local control. These patients may benefit from further dose escalation, since the liver function is not compromised by long-term exposure of the liver to viral hepatitis or alcohol consumption.

Use of SBRT for HCC is often initiated only after multiple attempts for local control in curative as well as palliative settings [6]. Many studies have shown the efficacy of SBRT in local control for HCC. Although it seems obvious that patients with multi-segmental recurrences are prone to intrahepatic out-field failures, this is the first prospective study to show that these patients may not be good candidates for SBRT. We suggest that a history of multi-segmental recurrences may need to be an exclusion criterion if SBRT is to be used with a curative aim. Another approach may be to use SBRT in combination with transcatheter arterial chemoembolization (TACE). SBRT may be used for intrahepatic tumors after incomplete treatment with TACE [24], or in combination with TACE or sorafenib for the treatment of multiple HCCs.

The current study is limited by inclusion of mostly small tumors (range 1.0–3.3 cm), and the SBRT protocol needs to be verified for larger tumors. A phase II trial to determine the efficacy of the current SBRT protocol is underway.

Helical IMRT-based SBRT was well-tolerated and showed promising results for adequately selected HCC patients. Exclusion of patients with multi-segmental recurrence prior to SBRT may improve out-field intrahepatic and extrahepatic failure rates.

## MATERIALS AND METHODS

### Study end points and eligibility

The primary end point was toxicity assessment, and the secondary end point was local control rate. A diagnosis of HCC was based on either pathologic confirmation or radiologic findings with an elevated serum level of alpha-fetoprotein (AFP) (> 400 ng/mL) in patients with a high risk of developing HCC [25].

### Eligibility

The study protocol conforms to the ethical guidelines of the 1975 Declaration of Helsinki, and was approved by our Institutional Review Board (4-2011-0650). All subjects gave informed consent prior to enrollment. All enrolled cases were presented at a multidisciplinary tumor board at our institution. Inclusion criteria were as follows: primary HCC not suitable for surgery because it was technically or medically inoperable or because of the patient's refusal; recurrence after multiple treatment including TACE and RFA; maximum tumor diameter ≤ 5 cm for a single tumor or the sum of diameters being ≤ 6 cm for up to 3 lesions; normal liver volume greater than 800 cm^3^; tumor located at least 1 cm from the wall of the stomach and/or bowel; no prior radiation therapy to the targeted area; adequate liver function (total bilirubin levels < 3 mg/dL, albumin levels > 2.5 g/dl, normal prothrombin time (PT) / partial thromboplastin time (PTT), and serum levels of aspartate aminotransferase (AST) and alanine aminotransferase (ALT) less than 3 times the upper limit of normal); adequate renal function (creatinine levels < 1.8 mg/dL or creatinine clearance > 50 mL/min); adequate hematological function (absolute neutrophil count, ANC ≥ 1500/mm^3^, platelet counts ≥ 50,000/mm^3^, hemoglobin level > 9 g/dL), no chemotherapy within 14 days of SBRT; Eastern Cooperative Oncology performance status 0–2; Child-Turcotte-Pugh's Class A or B; and age 20 years or older.

### Helical IMRT-based SBRT protocol

**Figure 4:**
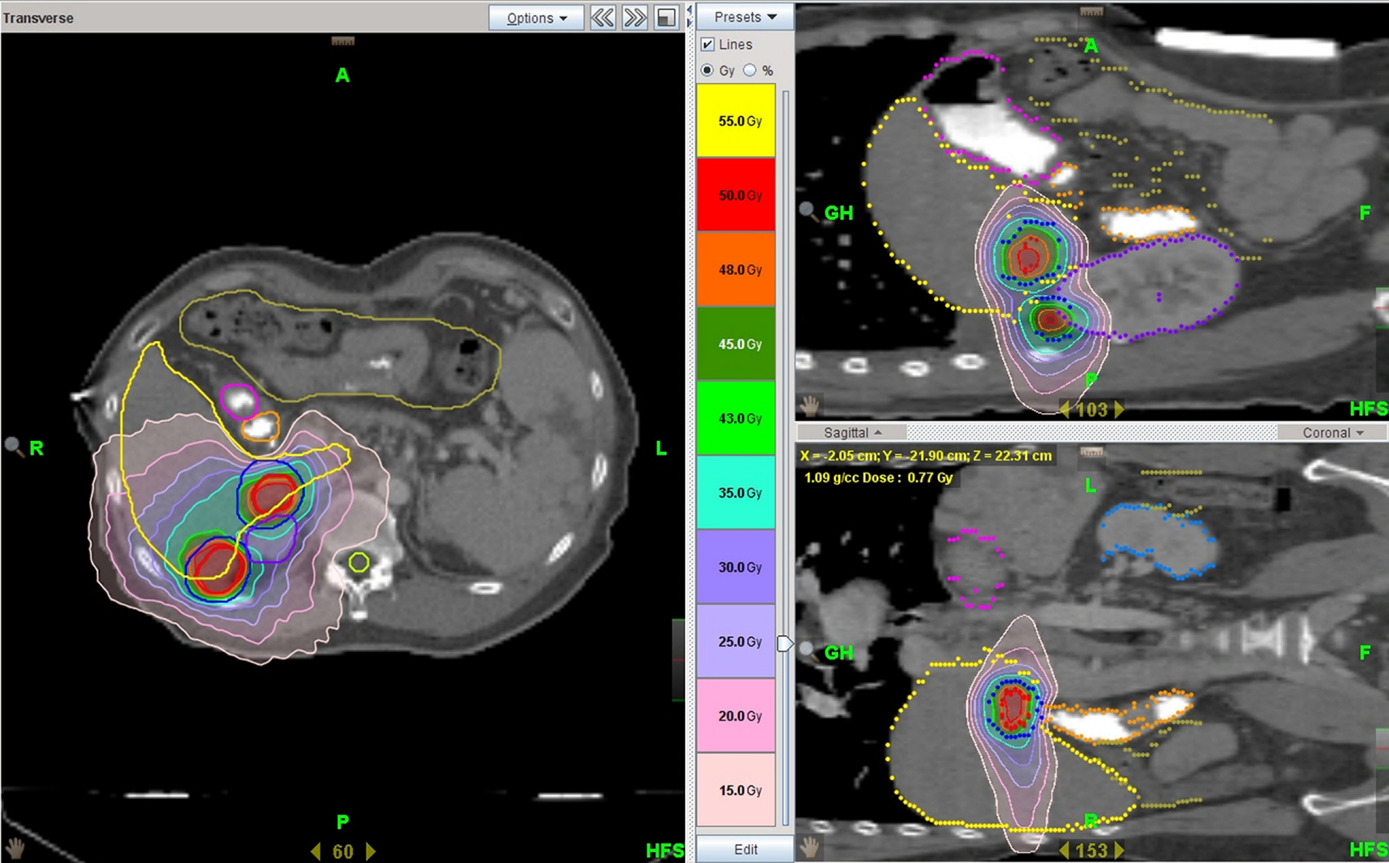
Target volumes and dose distribution for helical IMRT based-SBRT ITV is shown as a solid red line and PTV as a solid blue line for two target lesions.

Patients were immobilized using Vac-Lock^™^ (CIVCO, Coralville, Iowa) and an abdominal compression device was used to minimize internal organ motion. CT images were acquired over ten respiratory phases, with 1.5 mm slice thicknesses, under shallow respiration using a 4-dimensional CT simulator (SOMATOM Sensation, Siemens, Munich, Germany). Simulation CT images were fused with images from dynamic CT and MRI in order to aid accurate target delineation. The gross tumor volume (GTV) included all detectable tumors, as determined by dynamic CT and MRI. Internal target volume (ITV) was obtained by summing the GTVs of all respiratory motion phases. In order to incorporate microscopic satellite lesions into the target volume, CTV was defined as ITV plus a 5 mm margin in all directions. A radial margin of 5 mm and a craniocaudal margin of 7 mm were added to the CTV in order to define the planning target volume (PTV) (Figure 4). Helical IMRT-based SBRT planning was performed using a Hi-Art TomoTherapy Planning System (Accuray Inc., Madison, WI). The primary IMRT objectives were to maximize the dose to 95% of the PTV. Dose constraints included: at least 700 cc of total uninvolved liver receiving less than 15 Gy, less than or equal to 2/3 of the right kidney receiving greater than 15 Gy, maximum spinal cord dose 18 Gy, and maximum dose to the stomach or bowel 24 Gy. All patients were treated every-other-day with on-board MVCT for image-guidance [20]. The entire liver was scanned with MVCT immediately before each fraction of SBRT. The MVCT was aligned with the planning kVCT, with special attention to ensure that tumor-containing hepatic segments were exactly matched. Three dimensional offsets, left-right (x-axis), craniocaudal (y-axis), and antero-posterior (z-axis) offsets were recorded.

### Use of MVCT for setup corrections

Two sets of MVCT scans, immediately before and after SBRT, were acquired for 14 patients (patients 5 to 18). Three dimensional offsets in the left-right (x-axis), craniocaudal (y-axis), and antero-posterior (z-axis) directions were recorded. Total displacement (R) was calculated using the equation:

$$\text{R}=\sqrt{X^{2}+Y^{2}+Z^{2}}$$ [9]

Hepatic segments were divided into 5 groups: segment 1 in Group I (*n* = 1), segments 2, 3, and 4 in Group II (*n* = 3), segments 5 and 6 in Group III (*n* = 1), and segments 7 and 8 in Group IV (*n* = 9) [20]. The mean pre- and post-SBRT total displacement (R) was 3.9 ± 1.2 mm and 1.1 ± 0.7 mm for Group I, 6.3 ± 2.8 mm and 1.5 ± 1.0 mm for Group II, 7.5 ± 1.3 mm and 2.6 ± 0.8 mm for Group III, 5.0 ± 2.0 mm and 1.6 ± 1.1 mm for Group IV, and 5.4 ± 2.3 mm and 1.6 ± 1.1 mm for all patients (Table 5). The MVCT offsets of pre- and post-SBRT are shown in Figure 3.

### Dose escalation

Dose escalation started at 36 Gy (9 Gy/fraction) delivered in four fractions for PTV with a subsequent planned escalation of 2 Gy/fraction per dose-level. Three patients had to be treated in each dose level with no dose limiting toxicity within 1 month after SBRT before escalation to the next level was permitted. If toxicity occurred, a minimum of six patients were treated at that level.

### Evaluation

Patients were assessed during SBRT and after completion of treatment at 1 month, every 3 months for the first 12 months, and every 6 months thereafter. Dynamic liver CT or MRI was performed at each follow-up. Toxicity was graded using the CTCAE version 3.0. Dose-limiting toxicity (DLT) was defined as grade 3 or greater hepatic, GI toxicity occurring within 1 month of SBRT, or radiation induced liver disease (RILD) requiring treatment in the absence of disease progression within 3 months of SBRT [26]. The Modified Response Criteria In Solid Tumors (mRECIST) was used to evaluate treatment response [27]. Local failure was defined as an in-field recurrence within the high-dose region (> 80% isodose volume), or mRECIST progressive disease. Out-field recurrence was categorized into intrahepatic and extrahepatic (distant metastasis) recurrences.

### Statistical analysis

Local failure-free (LFFS), outfield intrahepatic failure-free (OFFFS), distant metastasis-free (DMFS), progression-free (PFS), and overall survival (OS) were estimated using the Kaplan-Meier method. Survival rates were defined as the time between the last day of SBRT and the first event. Events were death from any cause for OS, death or tumor progression for PFS, and recurrences as defined above for LFFS, OFFFS, and DMFS. Cox regression analysis was used for multivariate analysis. *P* values less than 0.05 were considered significant.
